# Effect of platelet-rich and platelet-poor plasma on 3D bone-to-implant contact: a preclinical micro-CT study

**DOI:** 10.1186/s40729-021-00291-5

**Published:** 2021-02-18

**Authors:** Dandan Song, Sohaib Shujaat, Yan Huang, Jeroen Van Dessel, Constantinus Politis, Ivo Lambrichts, Reinhilde Jacobs

**Affiliations:** 1 OMFS IMPATH Research Group, Department of Imaging & Pathology, Faculty of Medicine, KU Leuven, Kapucijnenvoer 33, 3000 Leuven, Belgium; 2 Oral and Maxillofacial Surgery, University Hospitals Leuven, Leuven, Belgium; 3 West China College of Stomatology, State Key Laboratory of Oral Disease & National Clinical Research Center for Oral Disease, Sichuan University, Chengdu, China; 4 Department of Morphology, Biomedical Research Institute, Hasselt University, Diepenbeek, Belgium; 5 Department of Dental Medicine, Karolinska Institute, Stockholm, Sweden

**Keywords:** Platelet-rich plasma, Osseointegration, Bone-implant interface, X-ray micro-CT, Dental implants

## Abstract

**Background:**

Bone-to-implant contact ratio (BIC%) plays a critical role in secondary stability of osseointegrated dental implants. The aim of this study was to identify the correlation of 2D/3D micro-CT images with histology as a gold standard for evaluating BIC% and to investigate the influence of the platelet-rich plasma (PRP) and platelet-poor plasma (PPP) on 3D BIC% following delayed implant placement with delayed loading (DIP+DL).

**Methods:**

Nine beagle dogs were recruited. Following bilateral extraction of mandibular 3rd premolar, 4th premolar, and 1st molar, 54 screw-type titanium implants were inserted and randomly divided into one control and two test groups based on a split-mouth design. The control group involved DIP+DL (*n* = 18) and both test groups included DIP+DL with local application of PRP (*n* = 18) and PPP (*n* = 18). A BIC analysis was performed utilizing 2D histomorphometry and 2D/3D micro-CT. Following identification of correlation between histology and 2D/3D micro-CT images, a 3D micro-CT assessment of the 3D BIC% at three follow-up time-points (1, 3, and 6 months) was carried out for observing the influence of PRP and PPP on BIC.

**Results:**

The 2D micro-CT BIC% values revealed a strong positive correlation with histology (*r* = 0.98, *p* < 0.001) and a moderate correlation existed with 3D micro-CT (*r* = 0. 67, *p* = 0.005). BIC levels at 1 month and combined influence of PPP and PRP irrespective of time-points revealed significantly higher 3D BIC% compared to the control. However, a reduction in 3D BIC% was observed at the 3rd and 6th month. No significant difference was observed between both PRP and PPP.

**Conclusions:**

Both 2D and 3D micro-CT demonstrated a potential to be utilized as a complimentary method for assessing BIC compared to the histological gold standard. Overall, both PRP and PPP significantly facilitated bone healing and osseointegration with a higher 3D BIC at follow-up. However, their influence was reduced as the observation period was increased.

## Background

Implant osseointegration has been described as the simultaneous contact and distant osteogenesis at the implant surface and osteotomy wall leading to new bone synthesis and a direct bone-to-implant contact (BIC) without intervening connective tissue [1]. The amount of BIC is an important determinant for achieving optimal secondary implant stability [2]. Amongst different methods proposed in literature for evaluating BIC, the BIC ratio (BIC%) is one of the most commonly utilized objective methods for quantifying the extent of osseointegration following implant placement [3]. It measures the bone in-growth in the transitional region and is defined as the percentage of the bone in contact with the implant surface. The success of implant is ensured when the BIC% is maintained at a minimum of 50–65% [4, 5]. Thereby, the fundamental goal of implant therapy is to obtain a favorable BIC to prevent implant micro-mobility and for maximizing the implant survival rate.

The two most commonly utilized imaging modalities for evaluating BIC% in animal models include histomorphometry and micro-computed tomography (micro-CT) [6, 7]. Although histomorphometry is a reliable method and has been utilized as a gold standard for measuring 2D BIC%, information acquired through two-dimensional (2D) slices is deemed insufficient for representing the three-dimensional (3D) bone structure [8]. To overcome the limitations associated with histomorphometry, micro-CT has been recommended for assessing BIC% based on its rapidity, reproducibility, and non-destructiveness [9]. Most of the evidence focusing on micro-CT assessment of BIC% are pseudo three-dimensional (3D) in nature, as instead of analyzing the true reconstructed 3D image, the measurements are performed on 2D cross-sectional images [10, 11]. Although, cross-sectional images are pseudo 3D, still they form a basis for validating the accuracy of micro-CT cross-sectional measurements compared to a histological reference.

In literature, various parameters have been reported to influence the BIC levels, such as, implant type and size [12, 13], implant surface treatment [14], bone quality and quantity [15], implant loading conditions [16, 17], and forces exerted on implant [18, 19]. Attempts have been made for enhancing the BIC values by modifying the roughness, composition, and surface treatment of the dental implants. Recently, multiple growth factors, most commonly, platelet-derived growth factors have been utilized for enhancing the BIC [20]. Some studies have shown improved implant stability and earlier osteogenesis with plasma containing higher concentration of platelets [21, 22]. However, there is lack of evidence related to the influence of different platelet-derived plasma concentrations on the 3D BIC at follow-up.

Therefore, the following animal study was conducted to address two aims. The first aim was to identify the correlation of 2D/3D micro-CT images with histology as a gold standard for evaluating BIC%. The second aim investigated the influence of the platelet-rich plasma (PRP) and platelet-poor plasma (PPP) on 3D BIC% following delayed implant placement with delayed loading (DIP+DL) utilizing micro-CT.

## Methods

### Animal model and sample size

The protocol for this study was approved by the Bioethics Committee of Sichuan University (reference No. WCCSIRB-D-2014-010) and complied with the ARRIVE guideline for preclinical studies [23]. Nine beagle dogs (weight 14–17 kg, age 12–14 months) were recruited. An identical housing and feeding condition were executed for all the animals at the Experimental Animal Center of Laboratory of Biotherapy. The sample size was in accordance with the previous studies [24–26] and also based on a priori power analysis in G*power 3.1 at a power of 80% and 0.05 level of significance [27].

### PRP and PPP preparation

A volume of 5 ml venous blood was collected from the cephalic vein of one of the forelegs of each experimental dog. The samples were transferred into sterile tube containing 1 ml of sodium citrate which acted as an anticoagulant and placed into a centrifuge (Allegra X 30R centrifuge, CA, USA). A previously optimized double-centrifugation protocol was applied for the preparation of PRP and PPP [28]. The first centrifugation involved a relative centrifugal force (RCF) of 700 g for 8 min and a second one at a RCF of 1600 g for 8 min. Following separation, 1 ml of each PRP and PPP were extracted from the sample. Both PRP and PPP were stored at room temperature in a conventional shaker until use.

### Surgical procedure

Each dog was administered with a 1-week prophylactic antibiotic therapy (gentamycin sulphate 300 mg) both before and after surgery for preventing infection. All surgeries were performed under general (0.1 ml/kg xylazine hydrochloride) and local anesthesia (2–4 ml lidocaine 2% epinephrine). A bilateral extraction of mandibular 3rd premolar, 4th premolar, and 1st molar was performed. After 1-month healing time, 54 screw-type titanium dental implant with plasma-sprayed hydroxyapatite (HA) coating (3.3 mm Ø × 8 mm, cylindrical, non-submerged healing, BLB, China) were inserted at the healed extraction sites (*n* = 6 per dog). The implants were randomly divided into one control and two test groups (*n* = 18 per group) based on a split-mouth design. The control group involved DIP+DL (*n* = 18) and the two test groups included DIP+DL with local application of PRP (*n* = 18) and PPP (*n* = 18). Each implant in the test groups was dipped in PPP or PRP solution prior to insertion in the alveolus. Implants were inserted with a controlled insertion torque ranging between 30 and 35 N cm. All surgical procedures were performed by an experienced oral and maxillofacial surgeon who was blinded to the allocation process. A resin crown was fabricated and placed onto each implant following 1 month of treatment with a resin cement (RelyX, Unicem, RX, 3 M ESPE, St. Paul, USA) under halogen light-curing unit for 20 s. Afterwards, an articulating paper (20-μm thick, Accufilm II, RX, 3M ESPE, St. Paul, USA) was used to check the contacts and make occlusal adjustments of the prosthetic crown to prevent possible overloading with the opposing natural teeth.

### Three-dimensional micro-CT assessment

A high-resolution micro-CT (Quantum FX Caliper, Life Sciences, Perkin Elmer) was utilized for acquiring radiographic images of the mandibular bone blocks with implants from each animal and analyzing BIC% follow-up changes at 1- (T1), 3- (T3), and 6-month (T6) time-points. The acquisition protocol included 20-μm pixel size, 360° rotation, 90-kVp tube voltage, 160-μA tube current, 180-s scanning time, and a 20-mm^2^ field of view (FOV). All the images were manually reoriented along the long axis of the implant in axial, coronal, and sagittal plane utilizing Dataviewer software (Ver. 1.5.1.2; Bruker-CT; Kontich, Belgium). Thereafter, image processing was performed with CT Analyser software (version 1.16.1.0, Skyscan1272, Bruker Microct, Kontich, Belgium). The whole implant body was included in the volume of interest (VOI).

**Fig. 1:**
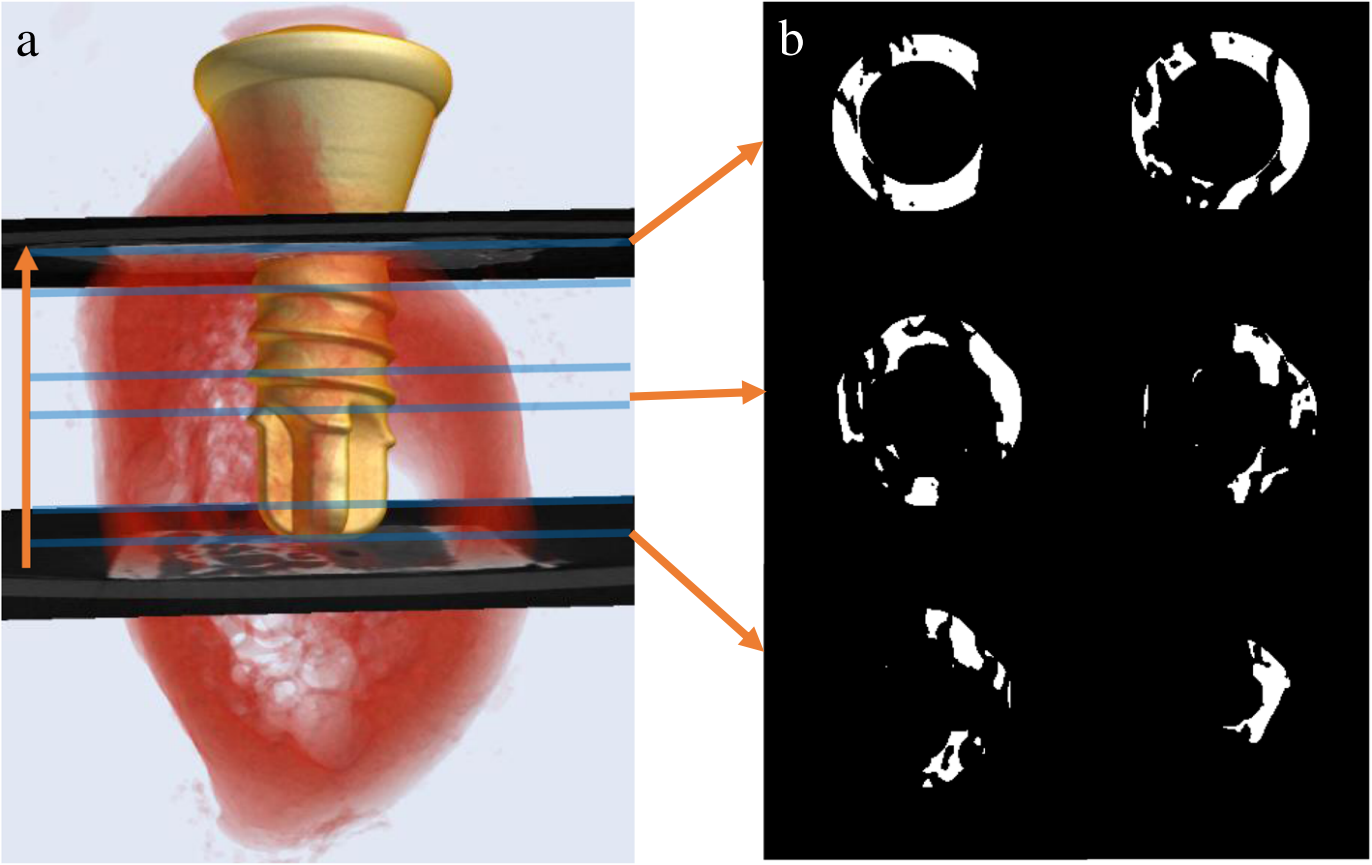
Automatic micro-CT-based evaluation of bone-to-implant contact (BIC) around the implant. **a** 3D surface reconstruction indicating the superior (implant shoulder) and inferior (implant apex) limit of volume of interest. **b** Binary images of BIC surface based on a custom processing with CTAn (version 1.16.1.0, Skyscan1272, Bruker Microct, Kontich, Belgium). Orange arrows indicate the series of slices from the implant apex to the shoulder level. Blue lines indicate the position of the images in **b** which show the amount of peri-implant bone descends from apex to coronal region

The implant shoulder was set as the reference for the superior limit of the VOI and the apex of the implant as the inferior limit (Fig. 1). The height of all the VOIs were kept constant for standardizing its vertical limit. Following determination of a circular region of interest (ROI) and binarization, a segmentation algorithm with separate histogram thresholding was applied for creating a distinction between implant (minimum, 43; maximum, 100) and bone (minimum, 110; maximum, 255) and calculating 3D BIC [29]. The BIC at a distance of 5 pixels from the implant surface was selected and BIC% was automatically calculated and represented by the percent intersection surface (I.S/TS) (Fig. 1).

### Histological assessment

Based on a random direct sampling technique, the dogs from each group were randomly euthanized at T1, T3, and T6 with an overdose of an intravenous injection of xylazine hydrochloride. Thereafter, bone blocks were prepared and decalcified with ethylenediaminetetraacetic acid (EDTA, 0.5 mol/L) and phosphate-buffered saline (pH 7.4) at 4 °C for 10 months. Care was taken to avoid damage to the samples while removing implants with surgical forceps. The bone blocks were dehydrated and fully infiltrated with paraffin and sliced into thin serial sections (~ 6 μm) in a bucco-lingual direction. All slices were stained with Masson’s trichrome stain. The BIC% was calculated based on a previously validated protocol [29].

**Fig. 2:**
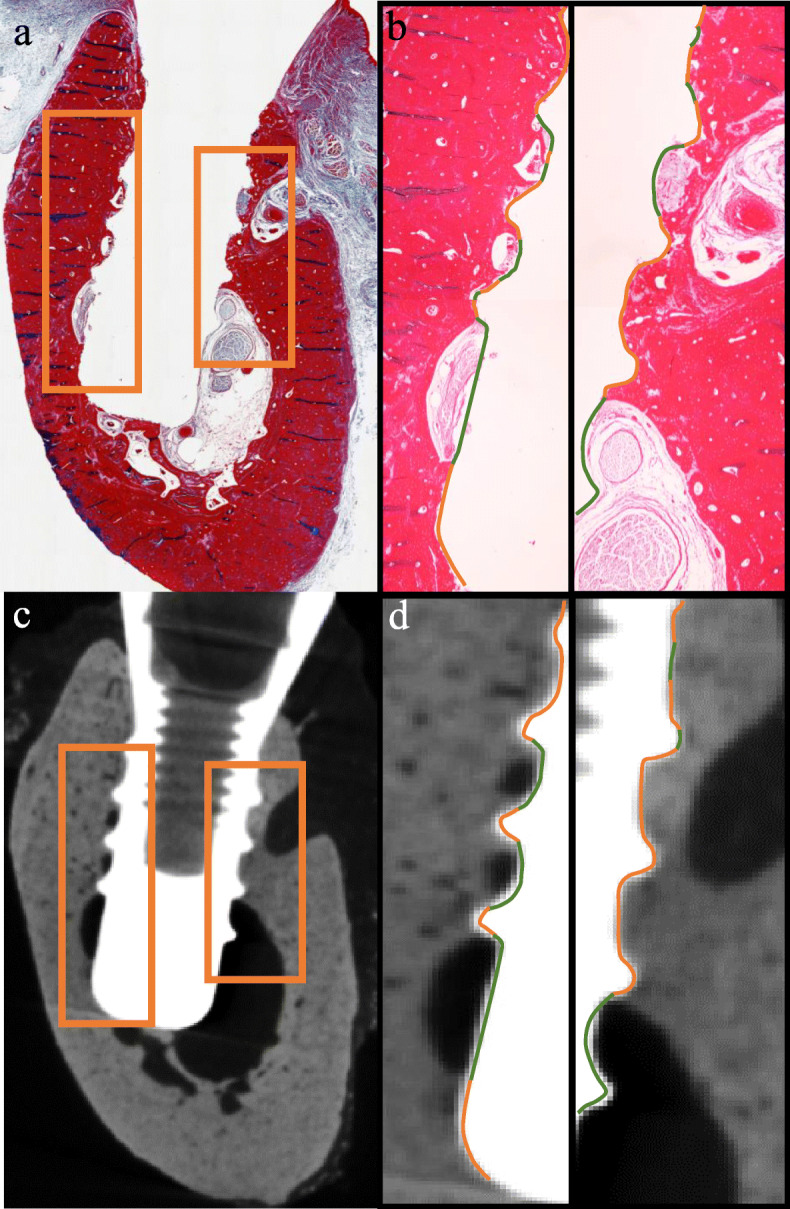
Matching sections of histology and micro-CT image and BIC% measurement. **a**, **c** Matching bucco-lingual images of histology and micro-CT. **b**, **d** Magnification of the orange rectangle in **a** and **c** (× 40). Orange line is the bone-to-implant contact; green line is the implant thread

Following selection of the matching histology slices and 2D micro-CT images, they were spatially aligned and registered for evaluating the same region as suggested by Soares et al. [30]. The BIC% was calculated by assessing the entire implant surface and contact areas between bone and implant using AxioVision software (Version 4.7.1; Carl Zeiss MicroImaging GmbH, Jena, Germany) (Fig. 2).

### Statistical analysis

The data analysis was performed using the SPSS software (Version 22, IBM, NY, USA). Normality of data distribution was assessed with the Shapiro-Wilk test. All the data showed normal distribution, thereby, parametric tests were utilized for assessing significance. The correlation between the 2D/3D micro-CT and histological BIC% was performed with the Pearson correlation coefficient. Afterwards, mean and standard deviation of the 3D micro-CT BIC% was calculated. Analysis of variance (ANOVA) and post hoc Bonferroni tests were employed for calculating the significance of difference between groups, different times points, and combined effect independent of time-points (*α* = 0.05).

## Results

All dogs remained in good health during the experiment. The implants in all groups were clinically stable till euthanasia with no sign of infection or any complication. The 2D BIC% values obtained from matching micro-CT and histology images revealed a strong positive significant correlation, regardless of the group or time-point (*r* = 0.98, *p* < 0.001). However, a moderate correlation existed between 3D micro-CT and histology images (*r* = 0. 67, *p* = 0.005).

Table 1 describes the 3D BIC% of the control and test groups at the 1st, 3rd and 6th month observation time-points. At the 1st month of healing time, PPP (*p* = 0.09) and PRP (*p* = 0.039) test groups showed the highest 3D BIC% with a significant increase compared to the control group. However, no significant increase was observed at the 3rd and 6th month. Both test groups showed a decline in 3D BIC% at the 3rd and 6th month compared to the 1st month, and the 6th month time-point showed a further decrease in 3D BIC% compared to the 3rd month without any significant difference. At the 6th month time-point, the PPP test group showed higher 3D BIC% (55.3 ± 8.7%) compared to PRP (52.9 ± 7.5%) and the control group (51.5 ± 4.7%) without any significant difference.

**Table 1:** 3D bone-implant contact ratio of control (DIP+DL) and test groups (DIP+DL+PPP, and DIP+DL+PRP) at different follow-up time-points using micro-CT (*p < 0.05)

Observation period	Group	Bone-implant contact, mean ± SD (%)	***p*** value; (1) vs (2), (1) vs (3), (2)vs (3)
1 month	(1) DIP+DL, (2) DIP+DL+PPP, (3) DIP+DL+PRP	52.2 **±** 3.2, 69.5 ± 2.6*, 73.4 ± 5.6*	0.09*, 0.039*, 1
3 months	(1) DIP+DL, (2) DIP+DL+PPP, (3) DIP+DL+PRP	47.3 ± 11.2, 59.0 ± 2.7, 65.2 ± 4.0	1, 0.513, 0.819
6 months	(1) DIP+DL, (2) DIP+DL+PPP, (3) DIP+DL+PRP	54.9 ± 7.7, 55.3 ± 9.1, 52.9 ± 7.1	1, 1, 1
Overall	(1) DIP+DL, (2) DIP+DL+PPP, (3) DIP+DL+PRP	51.5 ± 4.7, 61.3 ± 4.8, 63.8 ± 4.4*	0.078, 0.024*, 1

**Fig. 3:**
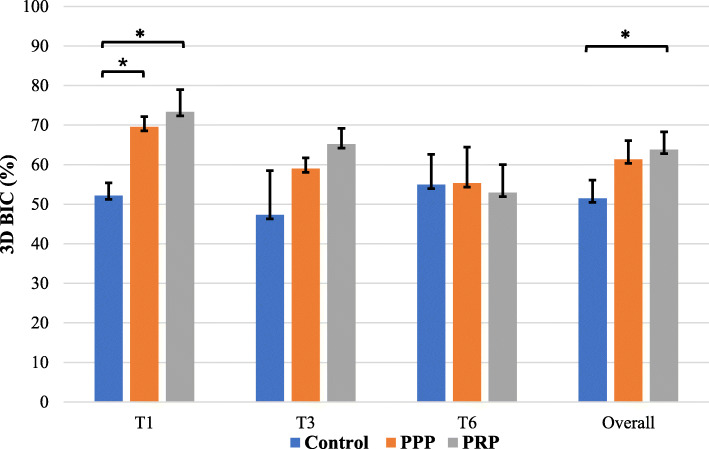
Impact of PRP and PPP on 3-demension bone-implant-contact ratio (3D BIC%) at individual time-points and overall influence irrespective of time. PPP, platelet-poor plasma; PRP, platelet-rich plasma; T1, 1 month; T3, 3 months; T6, 6 months. *p < 0.05 statistically significant

Overall, the 3D BIC% was found to be significantly high in PRP (63.8 ± 4.4%, *p* = 0.024) compared to the control (51.5 ± 4.7%) irrespective of the time-point (Fig. 3), with PRP group showing the most increase in 3D BIC%. No significant difference was observed between PPP and PRP group.

## Discussion

In our study, a strong correlation existed between 2D micro-CT and histology images, whereas with the utilization of 3D micro-CT, a moderate correlation was observed with a significant difference in BIC% of up to 4.9%. This minimal bias could be attributed to the absence of 3D BIC information in histology which varies depending on the implant surface being evaluated. Additionally, as the histological slices are taken at certain intervals at pre-selected locations which have a risk of misrepresenting, the entire osseous situation. Thereby, both 2D micro-CT and histological slices are unable to represent the true 3D bone structure around the implant. Our findings were consistent with prior studies which also indicated a strong correlation between 2D micro-CT and histology and a moderate correlation between 3D micro-CT and histology [11, 31]. For overcoming the limitations of 2D histological and pseudo 3D micro-CT images, we utilized a high-resolution 3D micro-CT-based methodology for calculating the 3D BIC% by excluding four pixels closest to the implant. These steps ensured standardized BIC% assessment without under- and over-estimation and overcame the influence of partial volume effect around the trabecular edges and beam hardening near the implant surface.

The 3D BIC% was assessed at different follow-up time-points following application of PRP and PPP compared to the control group. Our findings suggested a significant increase in BIC% at the 1st month following both PRP and PPP application, thereafter a slight decrease was observed at follow-up. Studies have shown significantly higher BIC% at an early healing phase following PRP application, which was in accordance with our findings [32]. This might be explained based on the short life-span of platelets (approximately 5–10 days) [33]. A high concentration of platelet growth factors is initially secreted within the first 10 min following blood clotting and within the first hour, over 95% of the presynthesized growth factors contained in the alpha-granules complete their secretion [34]. Following initial burst release of PRP-derived growth factors, they continue to synthesize and secrete additional growth factors during their remaining life-span [35, 36]. These growth factors promote and accelerate tissue healing and regeneration [37, 38]. Additionally, the centrifugated platelets in the form of PRP also accelerate cell proliferation and bone healing. However, no studies were found assessing the influence of PPP which requires further investigations. The long-term influence of plasma leading to significant enhancement of bone formation in intra-bony periodontal defects and sinus augmentation has been well-documented [39–41]. However, it remains unclear and controversial whether long-term effect of PRP and PPP can be regarded as clinically favorable or not in implant therapy.

When considering the overall combined impact of PPP and PRP irrespective of the time interval, an obvious increase in BIC% was observed with more than 60% in both test groups. Our findings were in an agreement with some studies [32, 42, 43]; at the same instance, inconsistencies were observed with other studies suggesting no influence of PPP or PRP on new bone formation [44–46]. The possible explanation for the inconsistent findings could be due to the different centrifugation techniques which can lead to substantial difference in platelet, leucocyte, and growth factors level. This requires further research to find an optimal standardized method for preparing plasma and later on studying which factors can lead to higher implant stability. The concentration of platelet in PRP (776.2 ± 144.0 × 10^9^/L) is almost 2000 times higher than that in PPP (4.8 ± 1.8 × 10^9^/L) [22]. However, in our study, both PRP and PPP have positive effect on promoting bone regeneration independent of the amount of platelet concentration. As the number of fibrin fibers is greater in PPP compared to PRP [47], which could explain the final biological bone healing effect of PPP during which lower platelet count is compensated by the fibrin fibers. The potential of these fibers acting to provide a scaffold for supporting cells and releasing growth factors should not be ignored. In some studies, PPP was discarded based on its low platelet nature [48, 49]. We believe, based on its potential in speeding up the bone regeneration regardless of the platelet concentration, future studies should also focus on the long-term impact of PPP on BIC.

The study had certain limitations. Firstly, primary implant stability was not evaluated. Secondly, the bone blocks underwent decalcification prior to histological preparation which might have impacted the BIC calculation, yet researchers carefully removed the implant after decalcification ensuring no further harm to the peri-implant bony surface. Thirdly, the small sample size could have led to bias within our findings. Future preclinical studies with a larger sample size should focus on quantifying the combined impact of different plasma preparation techniques, implant treatment protocols, bone density, and other modified implant surfaces on long-term 3D BIC%. Additionally, further animal research should be carried out assessing the influence of PRP and PPP following implant placement in compromised bone defects. Later on, the results of this analysis could then be applied and tested in patients with poor wound healing.

## Conclusions

Both 2D and 3D micro-CT demonstrated a potential to be utilized as a complimentary method for assessing BIC compared to the histological gold standard. However, further studies should be considered for improving the correlation of 3D micro-CT with histological images. Overall, both PRP and PPP were able to contribute towards a significantly higher BIC following implant treatment. However, their influence was reduced as the observation period was increased, with BIC having the highest ratio at the 1st month and lowest at 6 months. It can be assumed that the plasma-fibrin may facilitate and speed up bone healing and osseointegration.

## Data Availability

The datasets used and/or analyzed during the current study are available from the corresponding author on reasonable request.

## Abbreviations

BIC%: Bone-to-implant contact ratio
2D: Two-dimensional
3D: Three-dimensional
PRP: Platelet-rich plasma
PPP: Platelet-poor plasma
DIP+DL: Delayed implant placement with delayed loading
micro-CT: Micro-computed tomography
RCF: Relative centrifugal force
FOV: Field of view
VOI: Volume of interest
ROI: Region of interest
HA: Hydroxyapatite
